# The complete mitochondrial genome of freshwater snail, *Semisulcospira coreana* (Pleuroceridae: Semisulcospiridae)

**DOI:** 10.1080/23802359.2018.1443030

**Published:** 2018-02-23

**Authors:** Yi Kyung Kim, Sang Min Lee

**Affiliations:** Department of Marine Biotechnology, Gangneung Wonju National University, Gangneung, Korea

**Keywords:** Pleuroceridae, Semisulcospiridae, *Semisulcospira coreana*, mitochondrial genome

## Abstract

We have determined the mitochondrial DNA of *Semisulcospira coreana*. The complete mitogenome was 15,398 bp in length, encoding 13 protein-coding genes, 22 transfer RNA genes, and 2 ribosomal RNA genes. Phylogenetic analysis showed that the mitochondrial DNA of *S. coreana* was much closely related to other *Semisulcospira* species. This mitogenome could be helpful information for a variety of biodiversity researches in Korean freshwater snail.

The Semisulcospiridae freshwater snail is broadly distributed in much of Eastern Asia, including the Korea, southern China, and Japan (von Martens 1905; Davis 1969; Miura et al. 2013). Given its ecological or commercial importance, this species is considered as one of the valuable species in fisheries resources and become a potentially important target for aquaculture production in Korea (Kim et al. 2010). Using the mitogenome information, it could be applied to set up a specific barcode to assess genetic identification and diversity of Korean fresh snails or to provide essential information for the sustainable management of genetic resource.

The specimen was collected from Seomjin River (latitude: 35.1863844; longitude: 127.5263029) in Gurye-gun, Jeollanam-Do, South Korea and was stored in laboratory for Department of Marine Biotechnology, Gangneung Wonju National University, Gangneung, Korea. Total genomic DNA was isolated using the Exgene^TM^ Tissue SV DNA mini kit (Geneall, Seoul, Korea) following manufacturer’s instruction. We were sequenced and assembled using the Illumina Hiseq 4000 sequencing platform (Illumina, San Diego, CA, USA) and *SOAPdenovo* assembler at Macrogen (http://www.macrogen.com/kor/), South Korea, respectively. The accurate annotated mitochondrial genome was submitted to GenBank with accession number LC333861. The whole mitogenome sequence of *S. coreana* was 15,398 bp in length, consisting of 13 protein-coding genes (PCGs), 22 transfer RNA (tRNA) genes, and 2 ribosomal RNA (rRNA) genes. From the base composition analysis, as described for other gastropods (Zeng et al. 2015), an A + T bias was evident with an overall base composition of 30.95% for A, 34.68% for T, 16.19% for G, and 18.18% for C. Nine PCGs and seven tRNA genes were located in the heavy strand (H-strand), while four PCGs and 15 tRNA on the light strand (L strand). All PCGs were initiated with by the typical start codon (ATG), with exception (ATT) in nad5. Eleven PCGs (cox2, nad4l, nad4, nad5, cob, nad6, nad1, nad2, atp8, atp6, and nad3) were terminated with TAA stop codon and two PCGs (cox1 and cox3) had TAG as termination codon.

Phylogenetic tree was performed using MEGA 7 (ver.7.0.26) with maximum likelihood (ML) method (Kumar et al., 2016) (Figure 1). Topologies of the ML trees were evaluated by bootstrap test method with 1,000 replications under Kimura 2-parameter model. The mitochondrial DNA of *S. coreana* was much closely related to other *Semisulcospira* species and the *Cobitis choii* was used as an outgroup.

**Figure 1.**
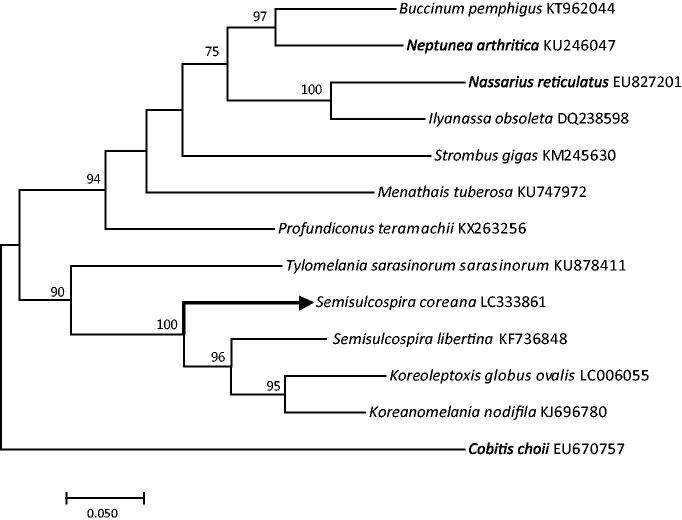
Phylogenetic relationship of *S. coreana* and 12 species beloning to Caenogastropoda based on cob sequences. Numbers in the nodes are the bootstrap values with 1000 replication.
